# Restoration of mitochondrial energy metabolism by electroconvulsive therapy in adolescent and juvenile mice

**DOI:** 10.3389/fpsyt.2025.1555144

**Published:** 2025-04-10

**Authors:** Ning Du, Yu Xie, Dandan Geng, Jingran Li, Heyan Xu, Yuna Wang, Jijia Gou, Xiwen Tan, Xiaoming Xu, Lei Shi, Yujia Chen, Fengming Chen, Zixuan Zhou, Gang Liu, Li Kuang

**Affiliations:** ^1^ Center for Mental Health, University-Town Hospital of Chongqing Medical University, Chongqing, China; ^2^ Medical Sciences Research Center, University-Town Hospital of Chongqing Medical University, Chongqing, China; ^3^ Department of Emergency and Critical Care Medicine, University-Town Hospital of Chongqing Medical University, Chongqing, China; ^4^ Psychiatric Center, The First Affiliated Hospital of Chongqing Medical University, Chongqing, China; ^5^ Department of Psychiatry, First Affiliated Hospital of Chongqing Medical University, Chongqing, China; ^6^ Sleep Medicine Center, Shiyan Hospital of Traditional Chinese Medicine, Shiyan, Hubei, China; ^7^ Department of Psychiatry, The First Clinical College of Chongqing Medical University, Chongqing, China

**Keywords:** electroconvulsive therapy (ECT), adolescent depression, mitochondrial energy metabolism, lactic acid, adenosine triphosphate (ATP)

## Abstract

**Background:**

Adolescent depression is an increasingly serious public health issue, and traditional treatment methods often have side effects or limited efficacy. Electroconvulsive therapy (ECT), a widely used treatment for severe depression, has recently gained attention for its potential in treating adolescent depression. Previous studies suggest that mitochondrial dysfunction is closely related to the onset of depression. Therefore, investigating the mechanism by which ECT alleviates depressive symptoms through the improvement of mitochondrial energy metabolism is of great significance.

**Methods:**

This study employed the chronic unpredictable mild stress (CUMS) mouse model to assess the effects of ECT on depression-like behaviors through the sucrose preference test, open field test, and tail suspension test. Additionally, mitochondrial energy metabolism markers, including ATP levels, oxygen consumption rate (OCR), lactate, and pyruvate, were measured in both mouse and human plasma to evaluate the effects of ECT on mitochondrial function.

**Results:**

The results showed that ECT significantly improved depression-like and anxiety-like behaviors in mice, as evidenced by the reversal of abnormal behaviors in the sucrose preference test, open field test, and tail suspension test. Analysis of plasma mitochondrial energy metabolism markers revealed that ECT significantly increased ATP levels, restored OCR, reduced lactate accumulation, and increased pyruvate levels. These findings suggest that ECT alleviates depressive symptoms by restoring mitochondrial energy metabolism and improving brain energy supply.

**Conclusion:**

This study systematically explored the potential mechanism by which ECT alleviates adolescent depression through the improvement of mitochondrial energy metabolism. The results indicate that ECT not only effectively alleviates depressive symptoms but also provides new insights and experimental evidence for the treatment of adolescent depression through mitochondrial function restoration. Future research could further investigate how to combine drug treatments to enhance mitochondrial function, improve ECT efficacy, and evaluate the effects of ECT in different depression subtypes, providing guidance for personalized clinical treatment.

## 1 Introduction

Major Depressive Disorder (MDD) in adolescents is a serious mental health condition characterized by persistent and widespread low mood, impaired cognitive function, and social dysfunction (1, 2). Adolescents with depression often experience significant health burdens, including academic decline, family conflicts, and an increased risk of suicide, which has become a leading cause of death in this age group (2). Unlike adult depression, the clinical manifestations and pathophysiological mechanisms of adolescent depression differ significantly in many aspects. Adolescence is a critical period for brain development, during which the maturation of the prefrontal cortex and emotion-regulating regions plays a crucial role in emotional regulation, decision-making, social behavior, and stress responses. The prefrontal cortex continues to mature during adolescence, and this area is closely related to functions such as emotional control, self-regulation, and social interaction. Despite advancements in pharmacological treatments and psychological interventions, a substantial proportion of patients remain resistant to conventional therapies, making the exploration of alternative treatment options an urgent need (3).

Electroconvulsive Therapy (ECT), a well-established and effective treatment for treatment-resistant depression, has demonstrated significant therapeutic effects in adolescents as well (4, 5). However, while the clinical efficacy of ECT has been widely validated, its underlying molecular mechanisms remain incompletely understood (6). Recent studies suggest that mitochondrial dysfunction—associated with disruptions in energy metabolism and oxidative stress—may play a pivotal role in the pathophysiology of depression (7). In this context, mitochondria serve not only as the cell’s “powerhouse” but also regulate oxidative balance, inflammatory responses, and neuronal survival, all of which are integral to the neurobiology of MDD (8, 9). Thus, understanding how ECT influences mitochondrial function could provide valuable insights into its therapeutic mechanisms, particularly in adolescents.

Although numerous studies have explored the neurobiological effects of ECT in adult populations, its impact on adolescents remains underexplored (10). Adolescence is a unique developmental stage marked by prominent synaptic plasticity, dynamic regulation of neurotransmitter systems, and ongoing neural circuit maturation (11). For example, mitochondrial dynamics—including biogenesis, fusion-fission balance, and mitophagy—exhibit distinct patterns during this developmental period, which may affect the body’s resilience or susceptibility to stress and treatment (12).ECT is believed to alleviate depressive symptoms by improving mitochondrial energy metabolism. ECT influences mitochondrial function through mechanisms such as promoting ATP synthesis, regulating oxidative stress levels, restoring mitochondrial membrane potential, and improving mitochondrial dynamics (7, 13–15). ECT can enhance the redox state of mitochondria, reduce oxidative stress, thereby alleviating neuronal damage, and restoring neuronal function (16–18).These neurobiological characteristics not only shape the presentation of mental health disorders in adolescents but also may influence the efficacy of treatments like ECT.

Moreover, mitochondrial dysfunction in peripheral tissues, such as blood, is increasingly recognized as a potential biomarker for brain metabolic disturbances (19, 20). Studies in adults have shown that ECT can regulate oxidative stress and mitochondrial respiration in peripheral tissue (14, 21). However, it remains unclear whether these effects extend to adolescents, or whether they correlate with improvements in depressive symptoms. Similarly, combining clinical studies with animal models, especially juvenile mice, to explore the underlying mechanisms could provide more direct insights into how ECT regulates mitochondrial function during this critical developmental stage. C57 mice, as a classic translational model, offer a valuable platform for examining the effects of ECT on both systemic and neural metabolic functions.

This study aims to investigate the effects of ECT on mitochondrial energy metabolism in adolescents with MDD and juvenile C57 mice, in order to determine whether ECT alleviates depressive symptoms by improving mitochondrial function. This study investigates clinical issues by establishing a mouse Chronic unpredictable mild stress induced (CUMS) depression model and applying Electroconvulsive Stimulation (ECS) intervention, aiming to gain deeper insights into the regulatory mechanisms of ECT on mitochondrial function. The findings provide a theoretical foundation for optimizing ECT treatment strategies for adolescent MDD patients and advancing the development of personalized therapeutic approaches.

## 2 Materials and methods

### 2.1 Clinical characteristics

This study recruited 30 hospitalized patients from the First Affiliated Hospital of Chongqing Medical University between January 2024 and July 2024. All patients met the following inclusion criteria: 1. diagnosed with unipolar depression by a psychiatrist according to the Diagnostic and Statistical Manual of Mental Disorders (DSM-IV-TR); 2. Hamilton Depression Rating Scale score of 17 or above; 3. no comorbid psychiatric disorders; 4. no systemic diseases, such as diabetes, hypertension, hyperthyroidism, or hypothyroidism; 5. aged between 13 and 18 years. Exclusion criteria: 1. failure to meet the inclusion criteria outlined above; 2. a history of epilepsy; 3. uncontrolled physical illnesses; 4. development of physical illnesses or refusal to undergo further ECT during the study. The study design was approved by the Ethics Committee of Chongqing Medical University(K2023-676). Written informed consent was obtained from all legal guardians and participants.

### 2.2 Electroconvulsive therapy

Prior to the initiation of ECT, each participant was provided with comprehensive information regarding the benefits and potential side effects of the treatment, followed by the signing of a written informed consent form. ECT treatments were administered between 8:00 AM and 12:00 PM using the Thymatron DGx system (Somatics, LLC, Lake Bluff, IL, USA), with electrodes placed bilaterally on the temples (23). Anesthesia and muscle relaxation were induced with propofol (1.5–2 mg/kg) and succinylcholine (0.5–1 mg/kg), respectively. The parameters were set as follows: pulse width 0.5 ms, frequency 30 Hz, energy percentage adjusted according to (age × 2/3), and bilateral electrode treatment. The initial energy setting is typically 50% of the estimated seizure threshold based on the patient’s age and is adjusted throughout the treatment course according to clinical response and seizure duration. The distribution of stimulation over time follows a standard clinical protocol, with patients receiving 6–8 ECT sessions within two weeks. The initial phase of treatment consists of 3–4 consecutive daily sessions, followed by treatments on alternate days. During ECT, the average seizure duration typically ranges from 20 to 40 seconds, monitored via EEG. A seizure is considered sufficient when its duration meets the minimum effective threshold for therapeutic response, and anesthetic dosage is adjusted as needed to optimize seizure quality. Following ECT, participants were strictly monitored for potential side effects, including dizziness, headaches, drooling, nausea, and vomiting. Concurrently, participants continued their prescribed regimen of antidepressant medications throughout the ECT treatment phase.

### 2.3 Blood samples

Blood samples were collected in the morning following a 12-hour fast, while participants were in the supine position, both at the beginning of the study and after four weeks. Five milliliters of venous blood were collected from each patient on ice with EDTA as an anticoagulant. Within 30 minutes of collection, the samples were centrifuged for 15 minutes at 1,000 g and 2-8°C. Subsequently, the isolated plasma was centrifuged at 2-8°C for 10 minutes at 10,000 g to ensure complete elimination of platelets.

### 2.4 Animal experiment design flow

**Figure 1:**
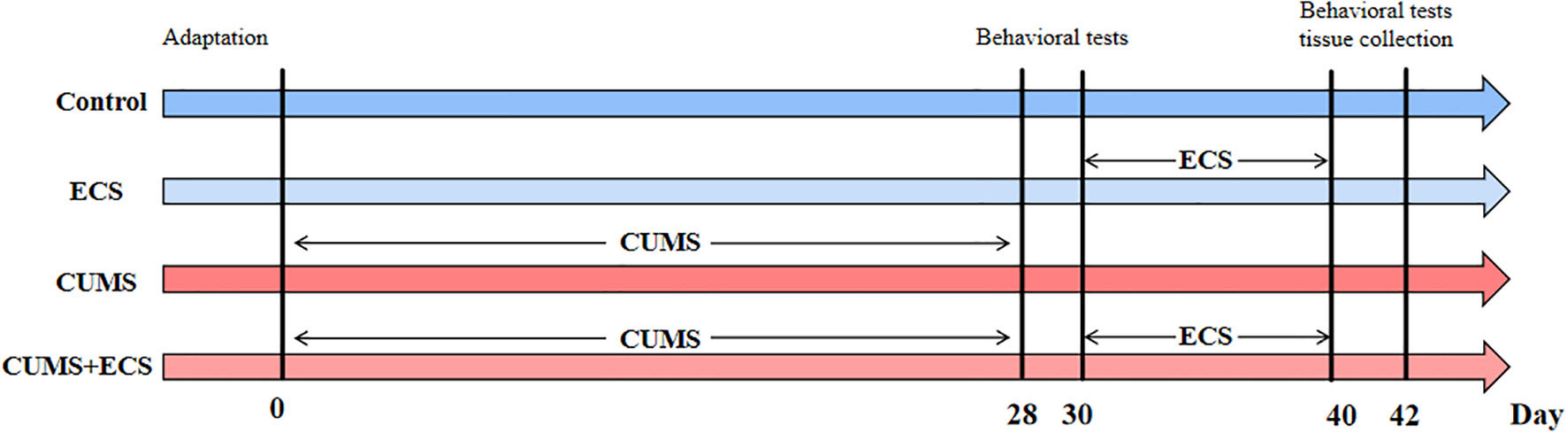
Animal experiment flowchart. ECS, Electroconvulsive Stimulation; Chronic unpredictable mild stress.

The animal experiment part of this study aims to investigate the effects of ECS on depression-like behaviors induced by CUMS in mice, as well as the associated behavioral and biomarker changes. The experiment consists of four groups: Control, ECS, CUMS, and CUMS + ECS. During the early phase of the experiment (Days 0 to 28), the CUMS and CUMS + ECS groups undergo chronic unpredictable mild stress intervention, using various stressors (such as water, light, and food deprivation) to simulate a prolonged stress condition, while the Control and ECS groups do not undergo any intervention. After Day 28, the ECS and CUMS + ECS groups receive ECS, while the CUMS and Control groups do not receive any sham ECS treatment. All groups undergo behavioral tests, including the sucrose preference test, tail suspension test, and open field test on Days 30 and 42, to assess the development and changes in depression-like behaviors. Additionally, plasma samples from the mice are collected to measure mitochondrial energy metabolism-related markers, in order to explore whether ECS improves depression-like symptoms through modulation of mitochondrial energy metabolism (**Figure 1**).

### 2.5 Establishment of a mouse CUMS-induced depression model

Mice were randomly assigned one week after conditioning, with no significant difference in body weight observed between the groups. Mice undergoing CUMS treatment were housed in isolation. The specific protocol for stress induction was appropriately modified based on previous literature (22, 23). Model mice were housed individually and subjected to a series of mild stress stimuli, including: long-duration: night strobe (12 hours), fasting (24 hours), restricted drinking (24 hours), wet pad (24 hours), empty pad (24 hours), tilted cage (24 hours), day-night (24 hours), long day (24 hours), long night (24 hours). Short-duration stimuli: restraint (3 hours), cold environment (4°C, 20 minutes), hot environment (36°C, 20 minutes), horizontal shock (shake, 30 minutes), tail clip (1 minute). Three different interventions were applied daily, with no intervention repeated within a five-day period, and the modeling lasted for four weeks. The control group received no treatment. The experimental design is shown in **Figure 1**.

### 2.6 Electroconvulsive intervention protocol for mice

The UGO-57800 electroconvulsive instrument (UGO BASILE, Italy) was used, with the following parameters: bidirectional rectangular wave, frequency 125 Hz, current strength 0.8 A, pulse width 1.5 ms, duration 0.8 s, and total output power of approximately 120 mJ. Mice in the normal and CUMS groups underwent sham-electroconvulsive treatment, with electrode pieces placed on both ears but no electrical stimulation for 15 seconds. Mice in the ECS and CUMS + ECS groups then received electroconvulsive treatment. The specific procedure was as follows: the mice were fixed on the operating platform in a prone position, with electrode pieces clamped to both ears, and were given 2 seconds of electrical stimulation. Electroconvulsive treatment induced strong tonic-clonic seizures in the mice. All mice were treated once daily, with treatments conducted between 2:00 PM and 5:00 PM for 10 days.

### 2.7 Animal behavioral experiments

#### 2.7.1 Sucrose preference test

Anhedonia, a core symptom of depression, was assessed using the SPT, a classic behavioral paradigm in rodents. A lower percentage of sugar-water preference indicates more pronounced depression-like behavior in mice. All mice underwent adaptive training prior to baseline testing for sugar-water preference. During the experiment, each mouse was housed individually and provided with two bottles of liquid: one containing plain drinking water and the other a 1% sucrose solution. The two bottles were randomly placed on the left or right side of the cage. After 24 hours, the remaining liquid was weighed and recorded. Both water and sucrose consumption were calculated, and the percentage of sugar-water preference was determined. The formula for calculating sugar-water preference percentage is: percentage = (sucrose consumption/(sucrose consumption + water consumption)) × 100%. The test was performed in a quiet and undisturbed environment.

#### 2.7.2 Open field test

The open field test (OFT) was used to assess the exploratory activity, anxiety, and autonomic behavior of animals in a novel environment. Reduced activity in the central area of the open field indicates higher levels of anxiety in mice. For testing, mice were gently placed in the center of a 50 cm × 50 cm × 50 cm open field box, which was divided into 25 squares: 9 squares in the central area and the remaining 16 squares as peripheral areas. Mice were allowed to move freely for 6 minutes, with activity in the central area recorded for 5 minutes using a video recording device.

#### 2.7.3 Tail suspension test

The tail suspension test (TST) was used to assess despair-like behavior in mice. In the experiment, mice were secured 1 cm from the tip of the tail and suspended 40 cm above the ground. Mice were placed 15 cm away from the nearest object and kept both visually and auditorily isolated. The test lasted for 6 minutes, and the immobility time after 5 minutes was recorded using a video recording device. Immobility was considered a manifestation of behavioral despair only when the mouse was completely stationary.

### 2.8 Mouse serum collection

Twenty-four hours after the behavioral experiments, 4 mice from each group were randomly selected and anesthetized by intraperitoneal injection of 0.4 mL/100 g of 1% pentobarbital sodium (Sigma, Germany). Once the mice were deeply anesthetized, the left thumb and index finger were used to compress both sides of the mouse’s neck, hindering venous return and causing the eyeball to protrude, indicating congestion of the retro-orbital venous plexus. A capillary tube (0.5×100 mm, cut into small pieces of approximately 3–4 cm before use) was placed at the medial canthus and inserted at a 30–45° angle relative to the plane of the nostrils, sliding behind the eyeball and gently piercing towards the fundus. When resistance was felt, the capillary tube was gently rotated while applying pressure to cut through the venous plexus, allowing blood to flow into the collection tube via capillary action (approximately 0.2 mL of blood). After blood collection, the mice were immediately euthanized by decapitation. The blood sample was left at room temperature for 1 hour, then centrifuged at 3000 rpm for 20 minutes at 4°C in a high-speed refrigerated centrifuge. After centrifugation, the serum remained at the top of the tube, with blood cells sedimented at the bottom. The serum was aspirated and aliquoted for storage at -80°C.

### 2.9 Quantification of plasma ATP, lactic acid, and pyruvate content

#### 2.9.1 Measurement of the ATP content

Measurement of the ATP Content An appropriate volume of plasma was diluted with an equal volume of double-distilled water, thoroughly mixed, and heated in a glass tube for 10 minutes. The mixture was then vortexed for 1 minute, centrifuged at 4000 rpm for 10 minutes, and the supernatant was collected for testing. Specific operations were carried out according to the manufacturer’s instructions (Nanjing Built Biological Engineering Research Institute, Nanjing, China).

#### 2.9.2 Determination of lactic acid and pyruvate content

Determination of Lactic Acid and Pyruvate Content The plasma samples were diluted 1:1 with normal saline and measured at a wavelength of 530 nm using a lactate measurement kit (Nanjing Built Biological Engineering Research Institute, Nanjing, China). The light absorption of pyruvate was measured at a wavelength of 530 nm using a pyruvate measurement kit (Nanjing Built Biological Engineering Research Institute, Nanjing, China).

### 2.10 OCR for the determination

Fresh brain tissue was carefully dissected, washed with cold PBS to remove excess blood, and then cut into thin slices. The tissue was digested with collagenase to obtain a single-cell suspension, and the cellular oxygen consumption rate (OCR) was measured using an oxygen consumption rate fluorescence kit (Elascience, China).

110 cells were seeded per well in a Seahorse XF Pro cell culture plate, and baseline OCR was recorded. OCR was then measured after the addition of 1 μmol/L oligomycin, 0.75 μmol/L FCCP, and 1 μmol/L antimycin A and rotenone, with results normalized to total cell protein concentration (unit: μmol/min).

### 2.11 Statistical analyses

Continuous variables following a normal distribution were analyzed using t-tests for statistical comparisons. For continuous variables not following a normal distribution, the Mann-Whitney U test was applied. The association between variables was explored using Pearson’s correlation test or Spearman’s rank correlation test, depending on the normality of the data distribution. A P-value of less than 0.05 was considered statistically significant. The analyses were conducted using IBM SPSS Statistics software, Version 26 for Windows.

## 3 Results

### 3.1 ECT treatment improves mitochondrial energy metabolism in plasma of depression patients

**Figure 2:**
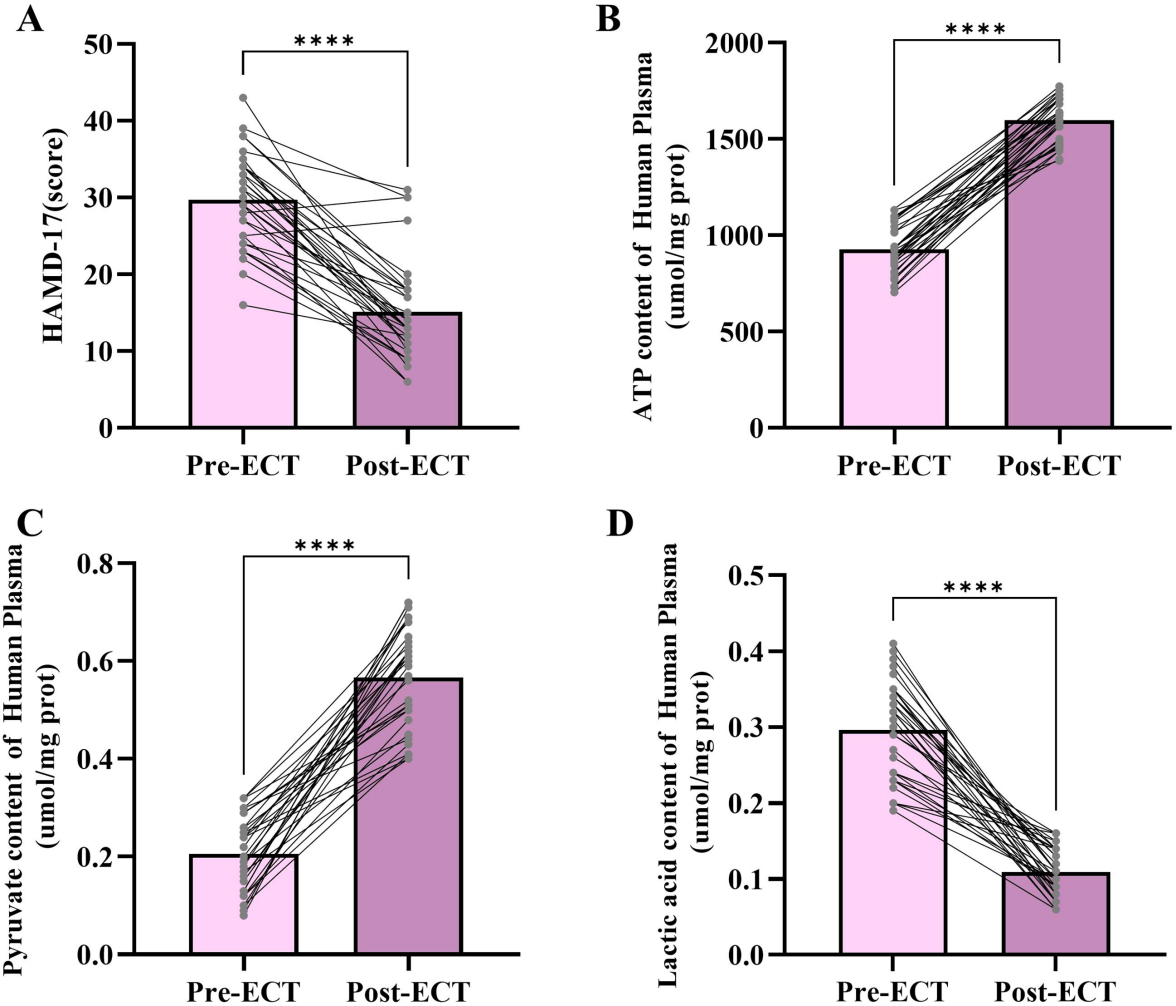
Changes in HAMD-17 scores, ATP content levels, pyruvate content levels, and lactic acid content levels following ECT. **(A)** HAMD-17 scores before and after ECT treatment. **(B)** Plasma ATP content before and after ECT treatment. **(C)** Plasma pyruvate content before and after ECT treatment. **(D)** Lactic acid content before and after ECT treatment. ****p < 0.001.

To further explore the relationship between adolescent depression and mitochondrial energy metabolism, we collected plasma samples from 33 adolescent depression patients before and after ECT treatment and measured changes in mitochondrial energy metabolism-related molecules in the plasma (**Figure 2**). The HAMD-17 score is widely used in clinical practice to assess the severity of depression. The results of the HAMD-17(t=9.402, df=64, p<0.001) assessment showed a significant decrease after ECT treatment, indicating that ECT treatment improves adolescent depression. The ATP (t=22.56, df=64, p<0.001) and pyruvate (t=16.20, df=64, p<0.001) levels in the plasma of adolescent depression patients significantly increased after ECT treatment, while lactate levels (t=14.94, df=64, p<0.001) significantly decreased. These results suggest that ECT treatment improves mitochondrial energy metabolism in depression patients.

### 3.2 CUMS can induce depression-like and anxiety-like behaviors in mice

**Figure 3:**
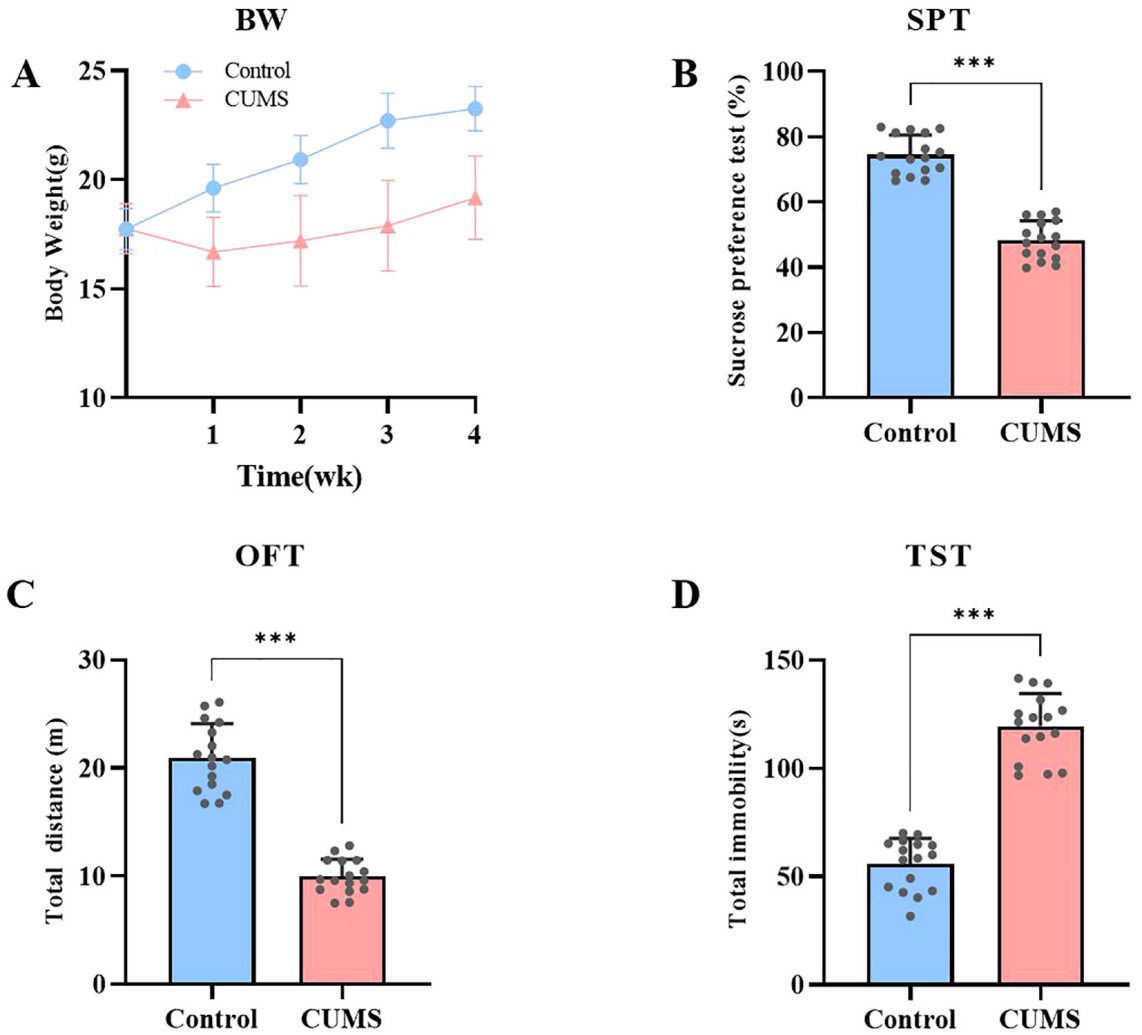
CUMS Induces Depressive-like and Anxiety-like Behaviors in Mice. **(A)** Body weight changes in both groups of mice during the modeling process. **(B)** Sucrose preference levels in both groups of mice. **(C)** Total distance traveled by both groups of mice in the open field test. **(D)** Immobility time of both groups of mice in the tail suspension test. Data are presented as mean ± SEM. ***p<0.001.

This study focuses on exploring depression using the CUMS model. Depression-like and anxiety-like behaviors were assessed by monitoring body weight changes, the sucrose preference test, the open field test, and the tail suspension test. After 4 weeks of CUMS exposure, the experimental group mice exhibited significantly more depressive-like and anxiety-like behaviors compared to the control group (p < 0.05).Body weight changes were continuously recorded throughout the modeling period. As the modeling progressed, significant differences in body weight were observed between the two groups. Control mice gradually gained weight over 4 weeks, following a normal growth trajectory, while weight gain in the CUMS group was significantly inhibited (**Figure 3A**). In the sucrose preference test, control mice displayed a normal hedonic response to the sucrose solution. In contrast, the percentage of sucrose preference in the CUMS group was significantly reduced (t=12.51, df=30, p < 0.001), indicating anhedonia, a core symptom of depression. This suggests that CUMS treatment led to the manifestation of significant depressive-like behavior in the mice (**Figure 3B**). In the open field test, the total activity distance of the control mice reflected normal exploratory behavior. The total activity distance decreased significantly (t=12.57, df=30, p < 0.001), indicating higher levels of behavioral inhibition and anxiety. This suggests that CUMS treatment led to a significant reduction in activity, reflecting stress-induced behavioral inhibition and anxiety (**Figure 3C**). In the tail suspension test, control mice exhibited normal struggling behavior. However, a significant increase was observed in the CUMS group (t=13.20, df=30, p < 0.001), indicating greater despair-like behavior. Despair-like behavior is one of the key behavioral indicators of depression (**Figure 3D**). These results indicate that ECS significantly improved the preference of mice in the sucrose preference test, markedly increased their activity levels in the open-field test and reduced the immobility time in the tail suspension test, demonstrating a significant alleviation of depressive symptoms induced by chronic stress.

### 3.3 ECS improves stress-induced depression-like behaviors in mice

**Figure 4:**
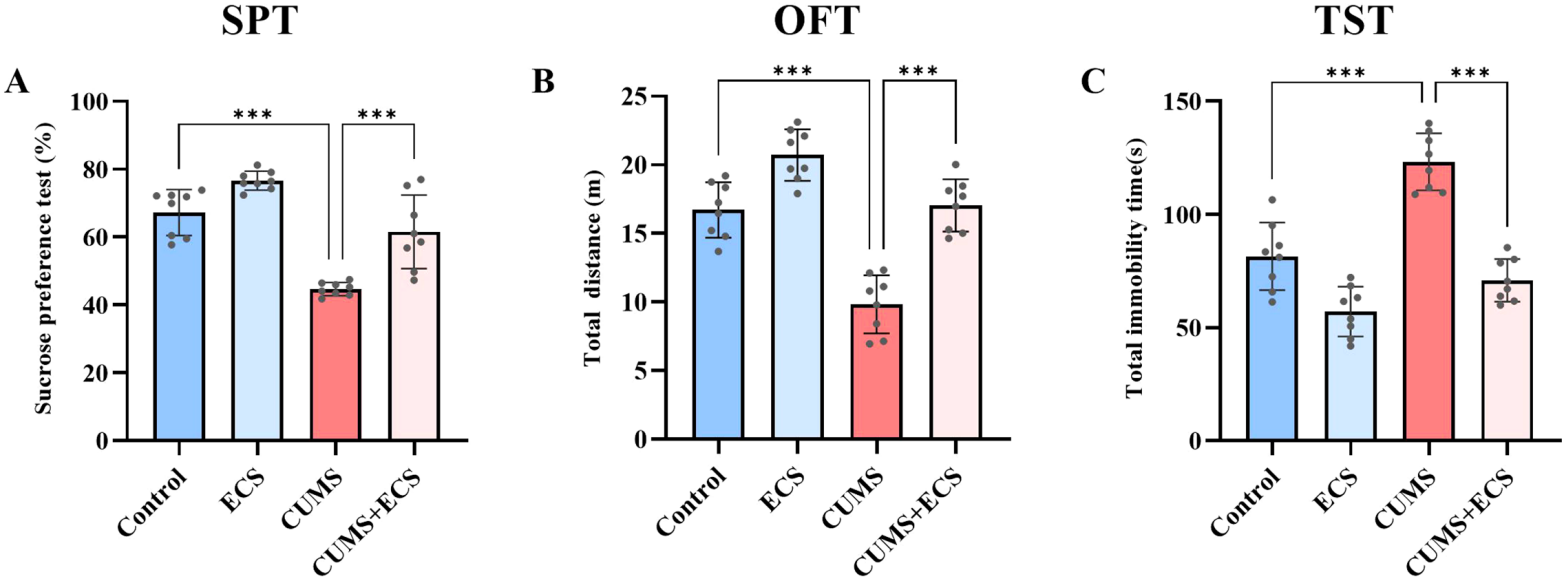
Effects of ECS on CUMS-Induced Depressive-Like Behaviors in Mice **(A)** The SPT levels in the four groups of mice after ECS. **(B)** Total distance traveled in the OFT by the four groups of mice after ECS. **(C)** Immobility time in the TST for the four groups of mice after ECS. Data are presented as mean ± SEM. ***P < 0.001.

This experiment investigated the effect of ECS on depression-like behaviors induced by CUMS. In the sucrose preference test, sugar water preference was slightly higher in the Control group compared to the ECS group. However, mice in the CUMS + ECS group showed a significant increase in sucrose preference compared to the CUMS group [F (3, 28) = 33.02, p < 0.001], indicating that ECS partially reversed the pleasure deficit induced by chronic stress (**Figure 4A**). In the open field test, the total activity distance of the ECS group was slightly different from that of the Control group. The total activity distance in the CUMS + ECS group significantly increased compared to the CUMS group [F (3, 28) = 41.90, p < 0.001], indicating that ECS partially restored the reduction in activity caused by CUMS (**Figure 4B**). In the tail suspension test, the total immobility time in the ECS group was slightly reduced but not significantly different from that of the Control group. A significant decrease in immobility time was observed in the CUMS + ECS group compared to the CUMS group [F (3, 28) = 43.99, p < 0.001], indicating that ECS partially reversed the increase in helpless behavior induced by chronic stress (**Figure 4C**). These results suggest that ECS can slightly improve behavioral performance in normal mice and significantly alleviate depressive symptoms induced by chronic stress.

### 3.4 ECS treatment regulates stress-induced mitochondrial energy metabolism in mice

**Figure 5:**
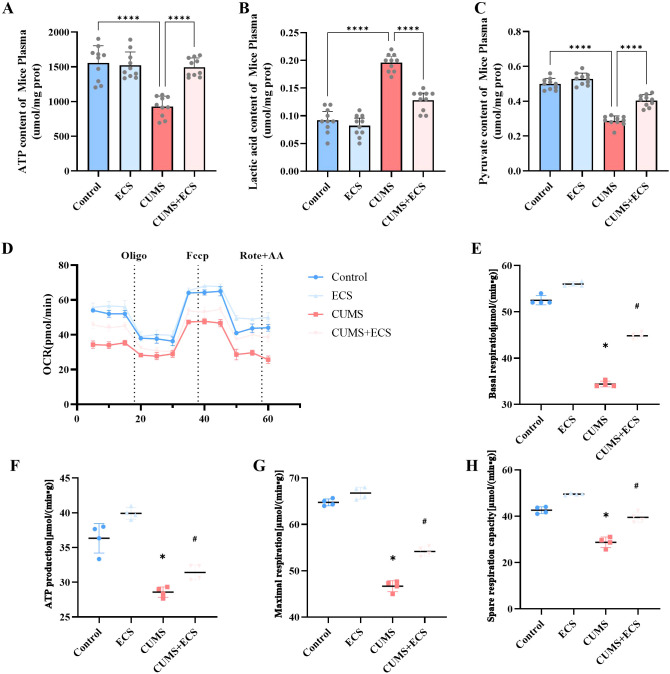
The regulation of mitochondrial energy metabolism in CUMS mice by ECS was assessed. **(A)** The ATP content levels. **(B)** Pyruvate content levels. **(C)** Lactate content levels. **(D)** Representative traces of the oxygen consumption rate. **(E)** Basal respiration. **(F)** ATP production. **(G)** Maximal respiration. **(H)** Spare respiratory capacity. Data are presented as mean ± SEM. *p < 0.05, #p < 0.05, ****p < 0.001. * indicates comparisons with the normal group; # indicates comparisons with the model group.

This experiment assessed the effect of ECS on mitochondrial energy metabolism in mice induced by CUMS. Mitochondria are the core of cellular energy metabolism, and ATP is the primary energy currency of the cell (24, 25). The ATP content in the CUMS group was significantly lower than in the Control and ECS groups. However, the ATP content in the CUMS + ECS group was significantly higher compared to the CUMS group [F (3, 36) = 26.10, p < 0.001, **Figure 5A**]. Lactate measurement results showed that the lactate content in the CUMS group was significantly higher than that in the Control group, and ECS treatment alleviated the lactate increase in the CUMS group [F (3, 36) = 74.18, p < 0.001, **Figure 5B**]. The pyruvate levels in the CUMS group were decreased, while ECS treatment significantly increased the pyruvate content in the CUMS group [F (3, 36) = 114.8, P < 0.001, **Figure 5C**]. OCR is commonly used as an important indicator for assessing mitochondrial activity and metabolic status (26). OCR measurement results showed that mitochondrial OCR in the CUMS group (**Figure 5D**), including baseline respiration [F (3, 12) = 695.9, p < 0.05, **Figure 5E**], ATP production [F (3, 12) = 59.11, p< 0.05, **Figure 5F**], maximal respiration [F (3, 12) = 301.9, p < 0.05,**Figure 5G**], and spare respiration [F (3, 12) = 97.18, p < 0.05, **Figure 5H**], were significantly reduced compared to the Control group. ECS treatment alleviated the effect of CUMS on OCR. These results suggest that ECS regulates mitochondrial energy metabolism in the brain tissue of mice.

## 4 Discussion

This study employed a periadolescent CUMS animal model with ECS intervention to investigate the role of ECT in improving adolescent depression, with a particular focus on validating the clinically observed ECT-induced lactate changes and their role in alleviating depressive symptoms. The experimental results showed that ECT significantly improved depressive-like and anxiety-like behaviors in mice, as assessed by the sugar-water preference test, open-field test, and tail suspension test. This finding is consistent with previous studies on the effects of ECT in animal models of depression (27, 28). Mice have a faster metabolism and neurophysiological changes compared to humans (29). Therefore, in animal experiments, daily ECS stimulation helps accumulate physiological changes more rapidly, simulating the long-term effects of ECT in humans and allowing observation of therapeutic effects within a limited experimental period (30, 31). In contrast, human ECT is administered less frequently (2–3 times per week for two weeks, totaling 6–8 sessions) (32, 33), aligning with a slower recovery process. Additionally, clinical ECT is constrained by safety and ethical considerations, as excessive frequency may increase the risk of side effects. Thus, a treatment schedule with sessions every other day or at longer intervals is typically adopted to ensure patient safety. Moreover, plasma mitochondrial energy metabolism markers measured before and after ECT treatment showed that ECT significantly increased ATP levels, restored oxygen consumption rate (OCR), reduced lactate accumulation, and elevated pyruvate levels. These findings indicate that ECT alleviates depressive symptoms by restoring mitochondrial energy metabolism, improving brain energy supply, and providing strong evidence for the use of ECT as a new treatment for adolescent depression.

From a clinical perspective, ECT is widely applied in the treatment of severe depression, bipolar disorder, and schizophrenia, especially when pharmacological treatments are ineffective or cause severe side effects (34–36). Previous research has confirmed that ECT exerts antidepressant effects by regulating neurotransmitter release, enhancing neuroplasticity, restoring neural network function, and improving neuronal metabolism (37, 38). This study further validates the antidepressant effects of ECT in a mouse model, providing experimental evidence for future clinical treatment of adolescent depression.

Glycolysis plays a pivotal role in brain energy metabolism and synaptic function, with its stability being essential for maintaining neuronal activity (39, 40).In patients with depression, impaired glycolysis leads to abnormalities in glucose metabolism, resulting in reduced ATP supply and energy deficits. This exacerbates neuronal dysfunction, ultimately impairing synaptic plasticity and worsening depressive symptoms (41, 42).Furthermore, accumulating evidence suggests that glycolytic dysfunction also disrupts mitochondrial metabolism, thereby compromising oxidative phosphorylation efficiency and limiting ATP production, accompanied by a decrease in the oxygen consumption rate (OCR). These metabolic impairments have been observed in the brains of individuals with depression (43–45).This evidence underscores the critical role of energy metabolism dysfunction as a key pathological mechanism underlying the onset and progression of depression. As the energy factory of cells, mitochondrial dysfunction not only leads to energy deficiency but also affects cell survival, proliferation, and synaptic plasticity, which can lead to mood disorders (46).ECT activates antioxidant enzymes, reduces oxidative stress levels, and decreases the production of reactive oxygen species (ROS), thereby protecting mitochondria from oxidative damage and maintaining their normal energy metabolism function. It may also increase mitochondrial membrane potential and restore the activity of ATP synthesis-related enzymes, enhancing ATP synthesis efficiency, providing sufficient energy for neurons, supporting their function, and improving their survival environment. Additionally, ECT regulates glycolysis by increasing glucose uptake and modulating the activity of related enzymes, promoting ATP production, especially under hypoxic conditions, providing energy support, improving cellular metabolism and energy supply, and further alleviating depressive symptoms. Previous studies have shown that ECT exerts antidepressant effects by regulating brain energy metabolism, primarily through enhancing oxidative phosphorylation and restoring mitochondrial function (7, 17, 47). The innovation of this study lies in its systematic exploration of how ECT alleviates adolescent depression by improving mitochondrial energy metabolism. Through behavioral tests and mitochondrial metabolic marker measurements, we demonstrated that ECT restored normal levels of ATP, OCR, and pyruvate in both mice and human plasma. This result is consistent with previous literature on ECT’s improvement of mitochondrial function (48), but this study provides a deeper analysis of the specific changes in mitochondrial energy metabolism before and after ECT treatment, revealing the potential mechanism by which ECT alleviates depressive symptoms.

Furthermore, this study employs a cross-species comparison by measuring mitochondrial metabolic markers in both mouse models and human plasma. Although research on ECT’s effects on human mitochondrial energy metabolism is limited, our study provides important preliminary data for this field. This cross-species comparison helps further validate the conclusions found in the mouse model and enhances the clinical relevance of the research. Unlike other studies, which mainly focus on adult or elderly depression patients, this study concentrates on adolescents, a group with unique physiological, psychological, and metabolic characteristics (10). Therefore, exploring the efficacy and mechanisms of ECT in this population is of great significance. Existing research has found that adolescent depression is closely associated with neurotransmitter imbalances, decreased neuroplasticity, and mitochondrial dysfunction during brain development (3, 49, 50). By examining changes in mitochondrial energy metabolism in an adolescent depression model, this study provides new insights and experimental evidence for clinical treatment in this population. Although existing literature suggests that ECT may treat depression by improving mitochondrial function (51), this study is the first to comprehensively assess the efficacy and mechanisms of ECT in adolescent depression, integrating behavioral performance and metabolic indicators, thus filling a research gap in this area. Our experimental data provide clear support for the hypothesis that ECT improves mitochondrial function and subsequently alleviates depressive symptoms.

This study has several limitations. It found that ECS can reduce lactate accumulation and improve mitochondrial energy metabolism; however, the causal relationship between lactate changes and the alleviation of depressive symptoms remains unclear. Lactate may be a consequence of improved mitochondrial function or may play a regulatory role during the ECS process, requiring further investigation. Additionally, this study primarily focused on the effects of ECS on mitochondrial energy metabolism, while ECT may exert its antidepressant effects through multiple mechanisms, such as regulating neurotransmitter release, enhancing neuroplasticity, and restoring neural network function. Future studies should incorporate molecular and imaging techniques to further explore these mechanisms.

Moreover, this study only evaluated the short-term effects of ECS and lacked long-term follow-up data, making it difficult to determine the long-term efficacy, potential side effects, and relapse risk of ECT. Furthermore, the small sample size and considerable individual differences among adolescents with depression—such as variations in disease course, genetic background, and metabolic state—may influence ECT efficacy. Additionally, the presence of uncontrolled concomitant pharmacological treatments in the study may impact the reliability and clinical generalizability of the findings. Future research should refine study designs to validate these conclusions more robustly.

The findings of this study not only provide experimental evidence for ECT’s alleviation of adolescent depression through improving mitochondrial energy metabolism but also offer new perspectives for clinical treatment. Mitochondrial dysfunction is considered a potential pathogenic mechanism of depression (52), and thus, mitochondrial-targeted therapeutic strategies may represent a novel direction for treating depression. Future research could explore how to enhance mitochondrial function through drugs or other interventions to complement ECT for better efficacy. Additionally, further studies should investigate the efficacy of ECT treatment in different depression subtypes and examine the influence of individual differences on ECT outcomes. More refined experimental designs focusing on specific ECT parameters could provide valuable guidance for personalized treatment in clinical practice.

## 5 Conclusion

This study shows that ECT alleviates depression-like behaviors in adolescent mice and humans by improving mitochondrial energy metabolism. ECT increased ATP levels, enhanced oxygen consumption rate (OCR), and reduced lactic acid accumulation. These results suggest that ECT imp0roves depressive symptoms by restoring mitochondrial function in the brain. ECT offers a promising treatment for depression, particularly in adolescents, and warrants further exploration to optimize its clinical use.

## Data Availability

The original contributions presented in the study are included in the article/supplementary material. Further inquiries can be directed to the corresponding authors.
